# The Association Between ABO Blood Groups and Neonatal Clinical Outcomes Among Infants Admitted to the Neonatal Intensive Care Unit of Imam Khomeini Hospital in Ahvaz, Iran, During 2023–2024: A Retrospective Descriptive Study

**DOI:** 10.1002/hsr2.73352

**Published:** 2026-10-07

**Authors:** Seyyed Mohammad Hassan Aletayeb, Masoud Dehdashtian, Mohammad Reza Aramesh, Arash Malakian, Amir Kamal Hardani

**Affiliations:** ^1^ Department of Pediatrics, Faculty of Medicine, Imam Khomeini Hospital Ahvaz Jundishapur University of Medical Sciences Ahvaz Iran

**Keywords:** diseases, major blood groups, neonates, outcome

## Abstract

**Background and Aims:**

Since the discovery of major blood groups, the potential relationship between blood groups and various diseases has garnered interest. Most studies in this area have focused on adults, with comparatively fewer related to children, particularly newborns. The aim of this study was to investigate the association between ABO blood groups and neonatal clinical outcomes.

**Methods:**

This retrospective descriptive study assessed the association between neonatal ABO blood groups and clinical outcomes among infants admitted to the Neonatal Intensive Care Unit of Imam Khomeini Hospital in Ahvaz, Iran, over 2 years (2023–2024). Infants were categorized by blood group (O vs. non‐O). Data on gestational age, delivery mode, intubation needs, sex, Apgar scores, birth weight, mechanical ventilation, proven sepsis incidence, surfactant use, antibiotic therapy, hospitalization duration, and mortality were collected and compared based on their blood groups.

**Results:**

Medical records of 545 infants were analyzed; blood group O was the most common (38.2%), followed by B (29.2%), A (23.5%), and AB (9.2%). Rh‐positive infants comprised 92.8% of the cohort. No statistically significant associations were found between blood group and gestational age, birth weight, Apgar scores, or intubation needs. However, male infants were significantly more prevalent in non‐O blood groups (60.5% vs. 39.5%, *p* = 0.001). Clinical outcomes, including surfactant use, mechanical ventilation, sepsis, and mortality, did not differ significantly between the two groups.

**Conclusion:**

The study highlights a sex disparity in blood group distribution but suggests that ABO blood type was not associated with differences in neonatal morbidity or mortality in unadjusted analyses in this population. Further research is warranted to explore underlying mechanisms.

## 1 Introduction

In 1900, Austrian physician Karl Landsteiner made a groundbreaking discovery about blood groups while at the University of Vienna [1]. Subsequent research demonstrated that the antigens and antibodies of the ABO blood group system are inherited traits [2]. Today, there are 47 recognized blood group systems, which encompass a total of 366 red blood cell membrane antigens, as identified by the International Society of Blood Transfusion (ISBT) [3].

The A, B, and H antigens of the ABO blood group system are found on the surfaces of red blood cells, epithelial cells, sensory neurons, vascular endothelial cells, and platelets [4]. For this reason, ABO antigens are referred to not only as blood‐group antigens but also as blood‐tissue antigens [5].

In adults, blood groups A, B, and AB have been linked to a higher occurrence of several types of cancer, including gastric, skin, pancreatic, lung, and ovarian cancers, as well as cardiovascular and respiratory issues such as venous and arterial thrombosis, myocardial infarction, ischemic stroke, and acute respiratory distress syndrome (ARDS) [6]. In studies that have examined the association of blood types with diseases or clinical conditions in children, researchers have reported that children with blood types A and AB were significantly more vulnerable to rotavirus gastroenteritis than children with blood type O [7]. Another study conducted in India showed that children with blood type O are more likely to contract dengue disease [8]. Boral et al. in 2013 found a significantly increased risk of sepsis with Enterobacter cloacae etiology in infants with blood type O compared to those with non‐O blood type [9].

On the other hand, a study conducted in China to investigate the relationship between pain after adenotonsillectomy in children with different blood types showed that children with blood types B and AB required significantly more fentanyl for pain than children with blood types A and O [10].

Timothy M. Bahr and colleagues conducted a multicenter study involving infants hospitalized in the neonatal intensive care unit (NICU) with refractory thrombocytopenia who had received more than 25 platelet transfusions. They found that all eight cases studied had blood type O. The authors suggest that thrombocytopenia in individuals with blood type O might have a worse prognosis [11]. In a retrospective multicenter study conducted by Yi Guan and colleagues, the researchers investigated the efficacy of inhaled nitric oxide therapy in infants experiencing persistent pulmonary hypertension of the newborn (PPHN) and its correlation with ABO blood groups. The findings indicated that infants with blood group O responded more favorably to the treatment compared to those with blood groups A and B [12]. I. Martynov et al., in a prospective, population‐based cohort study aiming to identify risk factors of focal intestinal perforation (FIP) and necrotizing enterocolitis (NEC) and enable faster diagnosis and treatment, showed that the risk of surgical FIP and NEC is higher in those with blood group AB and lower in cases having non‐AB blood groups [13].

In 2017, McMahon et al. compared the incidence of neutropenia at birth, sepsis, respiratory distress syndrome (RDS), and retinopathy of prematurity (ROP) in infants with blood type AB with those having other blood types. They found a significantly higher risk in the AB group [14]. Although the association between maternal‐neonatal ABO incompatibility and neonatal hyperbilirubinemia is well established and has been extensively studied [15], considerably less attention has been given to the relationship between neonatal ABO blood group itself and broader clinical outcomes among infants admitted to the neonatal intensive care unit. The present study was therefore designed to examine this less‐explored relationship, focusing on clinical course, interventions, length of hospitalization, and mortality rather than hyperbilirubinemia, which has already been extensively characterized in the existing literature.

## 2 Methods

### 2.1 Study Design and Population

This retrospective descriptive study was conducted at the Neonatal Intensive Care Unit of Imam Khomeini Hospital, Ahvaz, Iran. We reviewed the medical records of all infants admitted over a 2‐year period (2023–2024). Newborns with major congenital anomalies, known syndromes, or chromosomal disorders were excluded from the analysis.

### 2.2 Cohort Derivation

During the study period (January 2023 to December 2024), a total of 623 infants were admitted to the Neonatal Intensive Care Unit. Of these, 78 infants were excluded for the following reasons:

- Major congenital anomalies or chromosomal disorders: 42 infants
- Incomplete blood group data: 18 infants
- Transfer from other hospitals without complete initial records: 12 infants
- Incomplete outcome data: 6 infants
- The final cohort comprised 545 infants (87.5% of all admissions).

### 2.3 Missing Data

- Gestational age: 0 missing
- Birth weight: 0 missing
- Apgar score: 0 missing
- ABO blood group: 0 missing (complete after exclusion)
- Length of hospitalization: 0 missing
- Outcome data: 0 missing

### 2.4 Data Collection and Variables

Demographic and clinical information obtained from the newborns' records included gestational age, delivery mode, sex, birth weight, diagnosis, treatment course, length of hospitalization, and clinical outcomes. Infants meeting the inclusion criteria were categorized into two groups: those with blood group O and cases with other major blood groups.

The study assessed associations between blood groups and various characteristics, including gestational age, birth weight, and sex. Additionally, clinical features were analyzed, such as the 5‐min Apgar score, the need for intubation in the delivery room, the requirement for mechanical ventilation, and the incidence of sepsis confirmed by a positive blood culture. Treatment data included surfactant and antibiotic administration, duration of hospitalization, and outcomes. Comparisons were made between the two groups.

Culture‐proven sepsis was defined as a positive blood culture obtained within 72 h (early‐onset) or after 72 h (late‐onset) of life, accompanied by clinical signs of systemic infection (temperature instability, feeding intolerance, lethargy, or hemodynamic instability) [16]. As this was a retrospective descriptive study, respiratory distress syndrome was not independently coded as a discrete diagnostic outcome in the dataset; instead, associated clinical‐therapeutic interventions — intubation in the delivery room, surfactant administration, and the need for mechanical ventilation — were recorded as indirect indicators of respiratory morbidity.

### 2.5 Study Outcomes

The study was designed as an exploratory, hypothesis‐generating investigation. No single primary outcome was pre‐specified. Instead, a composite set of clinical outcomes was evaluated, including:

- Respiratory outcomes: Surfactant administration, mechanical ventilation, and intubation in the delivery room
- Infectious outcomes: Culture‐proven sepsis and antibiotic therapy duration
- General outcomes: Length of hospitalization and in‐hospital mortality

These outcomes were selected to capture the major domains of neonatal morbidity and were treated as exploratory clinical outcomes rather than pre‐specified co‐primary outcomes. Because the study was designed retrospectively and no a priori power calculation was performed for any individual outcome, all findings should be interpreted as hypothesis‐generating.

Infants were categorized based on gestational age, blood groups, and birth weight as follows:

- Gestational age: Term (≥ 37 weeks) and preterm (< 37 weeks)
- Birth weight: Normal birth weight (≥ 2500 g) and low birth weight (< 2500 g)
- Blood group: A, B, AB, and O

### 2.6 Blood Group Typing

In our department, ABO and Rh blood group typing is routinely performed for all hospitalized newborns. Blood group and Rh determination were conducted in the hospital laboratory using antigen‐antibody agglutination tests. The Rh and ABO blood groups were identified by adding antisera (A, B, and D) to three drops of plasma from each sample. Agglutination reactions were assessed using antisera (ABO reagents, hematyping kit, IR Iran). To compare the distribution of ABO blood groups in the neonatal cohort with that of the general healthy population, data were referenced from a large‐scale study involving 29,922 blood donors in Khuzestan province [17].

### 2.7 Sample Size

Sample size was determined by cohort availability (retrospective design) rather than a priori power calculation.

### 2.8 Ethics Approval and Consent to Participate

The research was conducted with the approval of the Pediatrics Department and the Research Vice‐Chancellor of Ahvaz University of Medical Sciences and in accordance with the World Medical Association Declaration of Helsinki. Due to the retrospective nature of the study, the need to obtain informed consent was waived by the Medical Ethics Committee of Ahvaz Jundishapur University of Medical Sciences (IR.AJUMS.HGOLESTAN.REC.1403.099).

### 2.9 Statistical Analysis

All statistical analyses were performed using IBM SPSS Statistics for Windows, version 25.0 (IBM Corp., Armonk, NY, USA). The normality of distribution for continuous variables was assessed using the Kolmogorov–Smirnov test.

#### 2.9.1 For the Primary Comparison between O and Non‐O Groups

• Continuous variables that followed a normal distribution were expressed as mean ± standard deviation (SD) and compared using the independent‐samples Student's t‐test.

• Continuous variables that did not satisfy the assumption of normality were reported as median (interquartile range [IQR]) and compared using the Mann–Whitney *U* test.

• Categorical variables were summarized as frequencies and percentages and compared between groups using Pearson's Chi‐square test; where the expected cell count was fewer than five, Fisher's exact test was applied instead.

#### 2.9.2 For the Secondary Four‐Group Comparison (A, B, AB, O)

• Continuous variables that followed a normal distribution were compared using one‐way analysis of variance (ANOVA).

• Non‐normally distributed variables were compared using the Kruskal–Wallis test.

• When the overall comparison was statistically significant, post‐hoc pairwise comparisons were performed with Bonferroni correction for multiple testing.

• Categorical variables were compared using Pearson's chi‐square test; where the expected cell count was fewer than five, Fisher's exact test was applied instead.

To compare the distribution of ABO blood groups in the neonatal cohort with that of the general healthy population of Khuzestan province, the chi‐square goodness‐of‐fit test was employed, using data from a previously published large‐scale study of 29,922 blood donors as the reference distribution [17].

Due to the exploratory descriptive design and the absence of a pre‐specified primary outcome, no multivariable regression analysis was performed. All comparisons were unadjusted, and the results should be interpreted accordingly.

All statistical tests were two‐sided, and a *p*‐value of less than 0.05 was considered statistically significant a priori. For post‐hoc pairwise comparisons in the four‐group analysis, a *p*‐value < 0.0083 (0.05/6) was considered statistically significant after Bonferroni correction. Statistical reporting was conducted in accordance with the Statistical Analyses and Methods in the Published Literature (SAMPL) guidelines.

## 3 Results

### 3.1 Cohort Characteristics

Hospital records of 545 infants were reviewed. Among them, 475 infants (87.2%) were born via cesarean section, while 70 (12.8%) were delivered vaginally. There were 300 male infants (55%) and 245 female infants (45%).

The mean gestational age was 32.46 ± 3.59 weeks, and the mean birth weight was 1935 ± 767 g. Forty‐eight infants (8.8%) required intubation in the delivery room. An Apgar score of < 7 at 5 min following birth was reported in 103 infants (19%).

Surfactant was administered to 195 infants (35.8%). Positive blood cultures were observed in 108 infants (19.8%). The median duration of hospitalization was 7 days (IQR: 9). A total of 72 newborns (13.2%) died during hospitalization (Table 1).

**Table 1:** Clinical and Demographic Characteristics of the Subjects (*N* = 545).

Variable	*N* (%)
Gestational age	
< 37 weeks	448 (82.2)
≥ 37 weeks	97 (17.8)
Birth weight	
< 2500 g	369 (67.7)
≥ 2500 g	176 (32.3)
Apgar score at 5 min	
< 7	103 (18.9)
≥ 7	442 (81.1)
Length of hospital stay	
≤ 7 days	267 (49.0)
> 7 days	278 (51.0)
Sex	
Female	245 (45.0)
Male	300 (55.0)
Mode of delivery	
Vaginal	70 (12.8)
Cesarean section	475 (87.2)
Intubation in the delivery room	
Yes	48 (8.8)
No	497 (91.2)
Surfactant administration	
Yes	195 (35.8)
No	350 (64.2)
Mechanical ventilation	
Yes	110 (20.2)
No	435 (79.8)
Blood culture result	
Positive	108 (19.8)
Negative	437 (80.2)
Outcome	
Alive	473 (86.8)
Died	72 (13.2)

The most common cause of hospitalization was neonatal prematurity (194 infants, 35.5%), while the least common was persistent pulmonary hypertension of the newborn (15 infants, 2.7%). The most frequent blood group among the neonates was type O (208 infants, 38.2%), and the least frequent was AB (50 infants, 9.2%). The majority of infants had Rh‐positive blood types (506 infants, 92.8%), while 39 infants (7.2%) were Rh‐negative (Table 2).

**Table 2:** Frequency of Major Blood Groups and Rh in Studied Infants (*N* = 545).

Blood group & Rh	Number	Percent
Group O	208	38.2
Group B	159	29.2
Group A	128	23.5
Group AB	50	9.2
Total	545	100
Rh Positive	506	92.8
Rh Negative	39	7.2
Total	545	100

### 3.2 Primary Analysis: O versus Non‐O Comparison (Table 3)

**Table 3:** Comparison of clinical and outcome variables between neonates with blood group o and neonates with other blood groups (A, B, AB combined).

Variable	Blood group O (*n* = 208)	Other blood groups (*n* = 337)	OR (95% CI)	*p*‐value
Gestational age (weeks), median (IQR)	34 (4)	34 (4)	—	0.096
Birth weight (g), median (IQR)	2060 (980)	2060 (1130)	—	0.524
Length of hospital stay (days), median (IQR)	7 (9)	8 (10)	—	0.761
5‐min Apgar score, median (IQR)	8 (2)	8 (2)	—	0.536
Sex				
Male	96 (46.2%)	204 (60.5%)	0.56 (0.39–0.79)	0.001
Female (Reference)	112 (53.8%)	133 (39.5%)	1.00	
Delivery room intubation				
Yes	16 (7.7%)	32 (9.5%)	0.79 (0.42–1.49)	0.535
No (Reference)	192 (92.3%)	305 (90.5%)	1.00	
Surfactant administration				
Yes	79 (38.0%)	116 (34.4%)	1.17 (0.81–1.67)	0.409
No (Reference)	129 (62.0%)	221 (65.6%)	1.00	
Mechanical ventilation				
Yes	38 (18.3%)	72 (21.4%)	0.82 (0.53–1.27)	0.442
No (Reference)	170 (81.7%)	265 (78.6%)	1.00	
Blood culture result				
Positive	44 (21.2%)	64 (19.0%)	1.14 (0.74–1.76)	0.581
Negative (Reference)	164 (78.8%)	273 (81.0%)	1.00	
Status at discharge				
Died	30 (14.4%)	42 (12.5%)	1.18 (0.72–1.96)	0.517
Alive (Reference)	178 (85.6%)	295 (87.5%)	1.00	

*Note:* Continuous variables are presented as median (IQR) and compared using the Mann–Whitney *U* test. Categorical variables are presented as *n* (%) and compared using the χ^2^ test or Fisher's exact test. Bold *p*‐values indicate statistical significance (*p* < 0.05).

Abbreviations: CI, confidence interval; IQR, interquartile range; OR, odds ratio.

Univariate analysis comparing infants with blood group O to those with non‐O blood groups (A, B, and AB) did not demonstrate statistically significant differences with respect to gestational age (median 34 weeks in both groups; *p* = 0.096), birth weight (2060 g vs. 2060 g; *p* = 0.524), 5‐min Apgar score < 7 (*p* = 0.536), or the need for intubation in the delivery room (OR 0.79, 95% CI 0.42–1.49; *p* = 0.535).

Surfactant was administered to 79/208 (38.0%) of blood group O infants and 116/337 (34.4%) of non‐O infants (OR 1.17, 95% CI 0.81–1.67; *p* = 0.409). Mechanical ventilation was required in 38/208 (18.3%) of blood group O infants and 72/337 (21.4%) of non‐O infants (OR 0.82, 95% CI 0.53–1.27; *p* = 0.442). Culture‐proven sepsis occurred in 44/208 (21.2%) of blood group O infants and 64/337 (19.0%) of non‐O infants (OR 1.14, 95% CI 0.74–1.76; *p* = 0.581). Mortality occurred in 30/208 (14.4%) of blood group O infants and 42/337 (12.5%) of non‐O infants (OR 1.18, 95% CI 0.72–1.96; *p* = 0.517).

However, a statistically significant association was observed between blood group type and sex: the proportion of male infants was significantly higher in the non‐O blood group category compared with females (60.5% vs. 39.5%; OR 0.56, 95% CI 0.39–0.79; *p*= 0.001) (Table 3).

### 3.3 Secondary Analysis: Four‐Group Comparison (Table 4)

**Table 4:** Baseline characteristics, clinical course, and outcomes of neonates stratified by neonatal ABO blood group (four‐group analysis).

Variable	Group A (*n* = 128)	Group B (*n* = 159)	Group AB (*n* = 50)	Group O (*n* = 208)	*p*‐value
Birth weight (g), median (IQR)	2200 (1080)	2000 (1210)	2150 (1085)	2080 (980)	0.401*
Gestational age (weeks), median (IQR)	34 (4)	34 (5)	35 (4.2)	34 (3.75)	0.064*
5‐min Apgar score, median (IQR)	8 (2)	8 (3)	8 (1)	8 (2)	0.088*
Length of hospital stay (days), median (IQR)	7.5 (9)	8 (12)	8 (10)	7 (9)	0.812*
Sex					0.0093**
Male, *n* (%)	74 (57.8%)	100 (62.9%)	30 (60%)	96 (46.2%)	
Female, *n* (%)	54 (42.2%)	59 (37.1%)	20 (40%)	112 (53.8%)	
Intubation in delivery room, *n* (%)	12 (9.4%)	18 (11.3%)	2 (4%)	16 (7.7%)	0.456**
Surfactant administration, *n* (%)	49 (38.3%)	55 (34.6%)	12 (24%)	79 (38%)	0.318**
Mechanical ventilation, *n* (%)	29 (22.7%)	34 (21.4%)	9 (18%)	38 (18.3%)	0.762**
Sepsis, *n* (%)	20 (15.6%)	36 (22.6%)	8 (16%)	44 (21.2%)	0.502**
Mortality, *n* (%)	13 (10.2%)	25 (15.7%)	4 (8%)	30 (14.4%)	0.402**

*Note:* Post‐hoc pairwise comparisons with Bonferroni correction (significance threshold *p *< 0.0083) did not identify any single pairwise contrast that remained statistically significant; the contrast between blood group B and blood group O showed the strongest, though non‐significant, trend (adjusted *p *= 0.012). Bold *p*‐values indicate statistical significance (*p *< 0.05).

* Kruskal‐Wallis test.

** Chi‐square test.

In the secondary analysis comparing all four blood groups, the results were similar to the two‐group analysis for most clinical variables. The sex distribution differed significantly across blood groups (*p *= 0.0093). However, post‐hoc pairwise comparisons with Bonferroni correction (significance threshold *p* < 0.0083) did not identify any single pairwise contrast that remained statistically significant. The contrast between blood group B (62.9% male) and blood group O (46.2% male) showed the strongest trend (adjusted *p*= 0.012), followed by blood group A (57.8% male) versus O (adjusted *p*= 0.298); blood group AB did not differ appreciably from O (60.0% male; adjusted *p*= 0.656). These findings indicate that the overall association between blood group and sex distribution (Table 4) reflects a broader pattern — blood group O infants having a lower proportion of males than the other groups combined — rather than any single significant pairwise contrast.

No other statistically significant differences were observed across the four blood groups for birth weight (*p *= 0.401), gestational age (*p *= 0.064), Apgar score (*p *= 0.088), length of stay (*p *= 0.812), surfactant administration (*p *= 0.318), mechanical ventilation (*p *= 0.762), sepsis (*p *= 0.502), or mortality (*p *= 0.402) (Table 4).

#### 3.3.1 Exploratory Subgroup Analysis: Very‐Low‐Birth‐Weight Infants (Table 5)

**Table 5:** Comparison of demographic, clinical, and outcome characteristics between very low birth weight infants with blood group O and non‐O blood groups (exploratory post‐hoc analysis).

Variable	Blood group O (*n* = 42)	Non‐O blood groups (*n* = 66)	OR (95% CI)	*p*‐value
Birth weight (g), median (IQR)	1040 (267)	1122 (287)	—	0.391
Gestational age (weeks), median (IQR)	28.5 (2.25)	29.4 (4)	—	0.311
5‐min Apgar score, median (IQR)	6 (3)	7 (4)	—	0.525
Length of hospital stay (days), median (IQR)	17 (26.25)	20 (27.25)	—	0.594
Sex				
Male	16 (38.1%)	38 (57.6%)	0.437 (0.198–0.968)	0.041
Female (Reference)	26 (61.9%)	28 (42.4%)	1.00	
Delivery room intubation				
Yes	12 (28.6%)	18 (27.3%)	1.067 (0.451–2.524)	0.883
No (Reference)	30 (71.4%)	48 (72.7%)	1.00	
Surfactant administration				
Yes	35 (83.3%)	41 (62.1%)	3.049 (1.177–7.897)	0.022
No (Reference)	7 (16.7%)	25 (37.9%)	1.00	
Mechanical ventilation				
Yes	20 (47.6%)	28 (42.4%)	1.234 (0.567–2.686)	0.597
No (Reference)	22 (52.4%)	38 (57.6%)	1.00	
Blood culture result				
Positive	22 (52.4%)	28 (42.4%)	1.493 (0.686–3.249)	0.313
Negative (Reference)	20 (47.6%)	38 (57.6%)	1.00	
Status at discharge				
Died	24 (57.1%)	27 (40.9%)	1.93 (0.93–4.01)	0.115
Alive (Reference)	18 (42.9%)	39 (59.1%)	1.00	

*Note:* Continuous variables are presented as median (IQR) and compared using the Mann–Whitney *U* test. Categorical variables are presented as *n* (%) and compared using the χ^2^ test or Fisher's exact test. Odds ratios (ORs) with 95% confidence intervals (CIs) were calculated using the second category as the reference. Bold *p*‐values indicate statistical significance (*p* < 0.05).

Abbreviations: CI, confidence interval; IQR, interquartile range; OR, odds ratio.

In an exploratory post‐hoc subgroup analysis of very‐low‐birth‐weight infants (n = 108), surfactant administration was significantly more frequent in the blood group O cohort compared to non‐O (83.3% vs. 62.1%; OR 3.049, 95% CI 1.177–7.897; *p*= 0.022). Given the post‐hoc nature of this analysis and the wide confidence interval, this finding should be considered exploratory and hypothesis‐generating. No other statistically significant differences were observed in this subgroup (Table 5).

## 4 Discussion

The association between major blood groups and the Rh factor with various diseases in both pediatric and adult populations has long been a topic of scientific interest. Early studies on ABO‐related susceptibility to COVID‐19 primarily focused on adults, but recent pediatric research has begun to fill this gap. Bari et al. examined 66 children in Pakistan with COVID‐19 or MIS‐C and found blood group A significantly linked to severe disease, despite a generally favorable prognosis in children [18]. Similarly, an Italian cohort study reported an association between blood group B and susceptibility to MIS‐C, along with rare immune‐related gene variants [19]. These findings indicate that ABO‐linked susceptibility in adults may not apply uniformly to children, highlighting the importance of group‐specific effects in this age group.

Our study revealed that the most common blood group among hospitalized neonates was group O, consistent with data from broader population studies conducted in our province and throughout Iran [20, 21]. Interestingly, blood group B emerged as the second most prevalent type in our cohort, contrasting with prior regional data that identified group A as the second most frequent. Although this discrepancy was not statistically significant, it highlights possible temporal or sampling variations or evolving demographic patterns.

The distribution we observed differs from the findings of Kayiran SM et al. in Türkiye and McMahon KE in the United States, where blood group A was reported as more common than B among newborns [14, 22]. The higher prevalence of group B in our cohort aligns with established geographic differences in blood group distribution across Middle Eastern and Central Asian populations, where blood group B is more frequently encountered than in Western countries [23]. This variation may be attributed to genetic drift, founder effects, or historical population movements.

In our study setting, the majority of infants admitted to the Neonatal Intensive Care Unit were born prematurely or with low birth weight. Others were full‐term but presented with complications such as respiratory distress, neurologic disorders, or nutritional challenges. Although our analysis found no statistically significant association between blood group and clinical characteristics in the overall cohort, the overrepresentation of blood group B prompts consideration of possible interactions with perinatal risk factors in specific populations.

Recent large population‐based studies further inform this discussion. Butler et al. found no association between maternal‐newborn ABO incongruence and bacterial infection risk within 30 days, though a protective effect emerged by 90 days [24]. A subsequent analysis of consecutive sibling births similarly found incongruence associated with lower infection risk, without cumulative maternal sensitization effects, suggesting maternal‐fetal ABO relationships may influence neonatal infection risk over longer time horizons than typically captured in NICU‐based studies like ours [25].

Interestingly, we identified a statistically significant sex‐related pattern: female neonates were more frequently associated with blood group O. In contrast, male neonates were more commonly found in non‐O groups. Biological sex is a well‐documented factor influencing immune response and disease risk. Hormonal differences — mainly estrogen and testosterone — modulate immune activity and may interact with ABO group‐related susceptibility to infection or inflammation [26, 27]. Furthermore, emerging research has begun to explore how epigenetic regulation and sex chromosome‐linked factors may influence ABO gene expression and downstream physiological effects [28]. In the analysis of the four blood groups, blood group O showed a numerically lower proportion of male infants than the other three groups combined, although no individual pairwise comparison reached statistical significance after Bonferroni correction; the contrast between blood group B and blood group O showed the strongest, though non‐significant, trend.

To date, there are no definitive studies linking blood group B to a higher risk of NICU admission or neonatal morbidity. However, the ABO blood group system has been involved in a variety of conditions such as thrombosis, cardiovascular disease, and certain infections in later life [25, 29, 30]. It remains plausible that ABO group antigens — known to modulate inflammatory and immune responses — may subtly influence neonatal outcomes. However, such effects might require larger sample sizes or molecular‐level analyses to detect.

The exact mechanisms underlying differences in disease presentation and severity among neonates of different ABO blood groups remain poorly understood. Genetic predisposition may play a role, including interactions between the ABO gene (on chromosome 9q34) and other regulatory genes affecting immune function and vascular biology [2, 4]. Additionally, maternal‐fetal ABO incompatibility and hemolytic disease of the newborn (HDN) are known concerns in perinatal medicine, although these conditions are usually diagnosed and managed early.

When comparing neonates with blood group O to cases having non‐O blood groups, our research could not find statistically significant differences in Apgar scores, birth weight, gestational age, need for delivery room interventions, respiratory support, surfactant therapy, incidence of culture‐positive sepsis, duration of antibiotic treatment, length of hospitalization, or mortality. These results align with those of Ufuk Cakir et al., who also reported no significant clinical differences based on blood group classification in unadjusted analyses [31].

Notably, among very‐low‐birth‐weight infants, surfactant administration was significantly more frequent in blood group O compared to non‐O infants (83.3% vs. 62.1%; OR 3.049, 95% CI 1.177–7.897; *p* = 0.022). The wide confidence interval reflects considerable statistical uncertainty, and the clinical relevance of this finding is limited by several factors. Surfactant administration is a therapeutic intervention that depends on clinician judgment and institutional practice patterns rather than a direct measure of respiratory severity. Gestational age, respiratory status at birth, antenatal steroid exposure, and other unmeasured confounders may influence its use. Accordingly, this finding should be interpreted as hypothesis‐generating rather than conclusive evidence of an association between ABO blood group and respiratory morbidity. Confirmation in larger, prospectively designed studies is needed before any causal or mechanistic inference can be drawn.

## 5 Limitations

This single‐center, retrospective design limits statistical power and generalizability. Maternal blood group data were not collected, representing a meaningful gap given the well‐recognized role of maternal‐neonatal ABO incompatibility in neonatal outcomes. Neonatal hyperbilirubinemia was intentionally excluded, as its association with ABO incompatibility is already extensively documented. Retinopathy of prematurity, intraventricular hemorrhage, and patent ductus arteriosus lacked systematic screening data, being assessed only when clinically indicated; bronchopulmonary dysplasia and necrotizing enterocolitis case numbers were too small for analysis. RDS was not independently coded as a discrete diagnosis; related treatment interventions served as proxies, potentially influenced by clinician discretion and institutional practice.

All reported comparisons were unadjusted. The term “independent” was not used in this study, as multivariable regression analysis was not performed. Potential confounders, including gestational age, birth weight, sex, and severity of illness, were not adjusted for in the statistical models.

Negative findings in this study should not be interpreted as evidence of no association. The confidence intervals for most effect estimates are wide and often cross the null value, indicating that smaller but clinically meaningful differences cannot be excluded. Larger studies with greater statistical precision are required to detect modest effects.

The VLBW subgroup analysis was conducted post‐hoc, prompted by the clinical observation that the overall cohort analysis did not demonstrate significant differences in most outcomes. This exploratory subgroup analysis was not pre‐specified.

Given the exploratory nature of this study and the absence of a single pre‐specified primary outcome, the sample size was determined by cohort availability rather than by a priori power calculation. Consequently, the study may have been underpowered to detect modest but clinically meaningful differences. Therefore, non‐significant findings should not be interpreted as definitive evidence of no association between ABO blood groups and neonatal outcomes.

Our findings are limited to infants already admitted to the neonatal intensive care unit. We cannot comment on whether ABO blood group influences the risk of NICU admission, as the study population does not include healthy infants born during the same period who were not admitted.

Future multicenter, prospective studies with standardized protocols and immunologic profiling are needed.

## 6 Conclusion

In summary, our findings suggest that the distribution of ABO blood groups among hospitalized neonates reflects that of the general population in the study region. Among neonates admitted to the Neonatal intensive care unit, no statistically significant associations were detected between ABO blood group and clinical course, complications, or outcomes in these unadjusted, exploratory analyses, except for a sex‐distribution difference primarily attributable to blood group B. The overall sex‐distribution difference across blood groups is not attributable to any single pairwise contrast after correction for multiple comparisons.

While these differences may not have immediate clinical implications, they highlight the need for further research into sex‐specific and genetic interactions related to the ABO system in neonatal health. Continued investigation with larger and more diverse populations is crucial to fully understand the potential impact of blood group antigens on neonatal clinical course, morbidities, and mortality.

## Author Contributions

**Seyyed Mohammad Hassan Aletayeb:** conceptualization, methodology, visualization. **Masoud Dehdashtian:** investigation, supervision. **Mohammad Reza Aramesh:** data curation, validation. **Arash Malakian:** writing – original draft, writing – review and editing, methodology. **Amir Kamal Hardani:** software.

## Funding

The authors have nothing to report.

## Ethics Statement

The research was conducted with the approval of the Pediatrics Department and the Research Vice‐Chancellor of Ahvaz University of Medical Sciences and in accordance with the World Medical Association Declaration of Helsinki. Due to the retrospective nature of the study, the need to obtain informed consent was waived by the Medical Ethics Committee of Ahvaz Jundishapur University of Medical Sciences (IR.AJUMS.HGOLESTAN.REC.1403.099).

## Conflicts of Interest

The authors declare no conflicts of interest.

## Transparency Statement

Arash Malakian, the corresponding author, affirms that this manuscript is an honest, accurate, and transparent account of the study being reported; that no important aspects of the study have been omitted.

## Data Availability

The data that support the findings of this study are available on request from the corresponding author. The data are not publicly available due to privacy or ethical restrictions. The data that support the findings of this study are not openly available due to reasons of sensitivity and are available from the corresponding author upon reasonable request. Data are located in controlled access data storage at the Research Office of Imam Khomeini Hospital, Ahvaz, Iran.
